# Development of a New Drill Design to Improve the Temperature Control during the Osteotomy for Dental Implants: A Comparative In Vitro Analysis

**DOI:** 10.3390/biology9080208

**Published:** 2020-08-06

**Authors:** Sergio Alexandre Gehrke, Raphaél Bettach, Benoit Cayron, Gilles Boukhris, Berenice Anina Dedavid, Juan Carlos Prados Frutos

**Affiliations:** 1 Biotecnos Research Center, Montevideo 11100, Uruguay; 2 Department of Biotechnology, Catholic University of Murcia, 30107 Murcia, Spain; 3 Department of Cariology and Comprehensive Care, New York University, New York, NY 10010, USA; rbettach@gmail.com; 4 Private practice, 77220 Gretz-Armainvilliers, France; 5 Private practice, 37000 Tours, France; cayron.benoit@gmail.com; 6 Private practice, 75012 Paris, France; boukhrisgilles@gmail.com; 7 Department of Materials Engineering, Pontificial Catholic University of Rio Grande do Sul, Porto Alegre 90619-900, Brazil; berenice@pucrs.br; 8 Department of Medicine and Surgery, Faculty of Health Sciences, Rey Juan Carlos University, 28933 Madrid, Spain; juancarlos.prados@urjc.es

**Keywords:** dental implants, drill design, irrigation system, osteotomy, thermocouple

## Abstract

The present in vitro study evaluated a new drill design to improve the temperature control during the osteotomies for dental implant installation, comparing with two drill designs that use conventional external irrigation. Three blocks of synthetic cortical bone were used for osteotomy procedures. Three groups were created: control group 1 (Con1), where a conical multiple drill system with a conventional external irrigation system was used; control group 2 (Con2), where a single bur with a conventional external irrigation system was used; and, test group (Test), where the new single bur (turbo drill) with a new irrigation system was used. Twenty osteotomies were made without irrigation and with intense irrigation, for each group. A thermocouple was used to measure the temperature produced during the osteotomies. The measured temperature were: 28.9 ± 1.68 °C for group Con1; 27.5 ± 1.32 °C for group Con2; 26.3 ± 1.28 °C for group Test. Whereas, the measured temperatures with irrigation were: 23.1 ± 1.27 °C for group Con1; 21.7 ± 1.36 °C for group Con2; 19.4 ± 1.29 °C for group Test. The single drill with a new design for improving the irrigation and temperature control, in comparison with the drill designs with conventional external irrigation.

## 1. Introduction

The osteotomy protocols, regardless of the system used, determine that it should be performed with a low-temperature variation, never exceeding 47 °C, as it could denature bone tissue proteins and generate necrosis in that area [1]. Several studies have been developed with different irrigation systems and with different drill designs to improve and decrease trauma during the osteotomy procedure for installing implants and, consequently, reducing inflammatory reactions [2,3,4].

Recently, Salles and Collaborators (2015) [5], reported through an experimental study using an immunohistochemical analysis for the inflammatory factor NFkB (nuclear factor kappa-light-chain-enhancer of activated B cells), that irrigation plays an important role in controlling this endonuclease and, obviously, in controlling the intensity of the inflammatory process. In this sense, other histological studies have also shown that the healing response of bone tissue around implants can be improved when using drilling systems designed to reduce the trauma caused during osteotomy procedures [6,7,8].

Regarding the instruments and techniques used to perform osteotomies for the installation of implants, different factors must be considered and analyzed, such as irrigation method (external or internal), drill design, drilling speed, the number of drills (single or multiples), drill material, drilling movement (continuous or intermittent), equipment used (rotary or oscillatory), force applied, among others. For these findings, the methods of evaluating the efficiency of the systems proposed for performing osteotomies, the most used is the evaluation of temperature control during the procedure. To perform these experiments, thermosensors installed near the location where the drilling or infrared sensors can be used, both of which have similar results, but may vary in practicality to the operator during the tests [2,4,9]. In addition, several substrates are used to perform this type of in vitro test, mainly, bone of animal origin and synthetic bone [10].

However, there is no consensus on the ideal system for osteotomy, both in terms of the number of drills, and in terms of their ideal design. In this sense, a device was developed and coupled to the drill shank, creating a new drill design, which has the function of boosting and directing the flow of the irrigating liquid into the bone tissue, thus increasing the effectiveness of refrigeration during the osteotomy procedure. Then, the present in vitro study evaluated this new drill design to improve the temperature control during the osteotomies, comparing with two drill designs (single and multiple sequence) that use conventional external irrigation.

## 2. Materials and Methods

**Figure 1:**
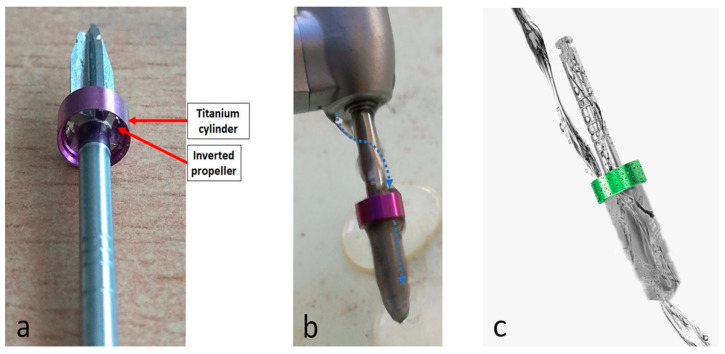
Representative image of the details of new drill design (**a**), that present an accoupled cylinder with an inverted propeller to improve the irrigation. (**b**) The blue arrows indicate the liquid is driven by the propeller down into the blades. (**c**) Schematic image of the path taken by the irrigating liquid.

In this study, two groups of drill systems with conventional external irrigation were used as control and, compared with the new drill design (TURBOdrill^®^, Implants Diffusion International, Montreuil, France) that features a device attached to its stem to boost and direct the flow of the liquid used for irrigation. This device featured a titanium cylinder with an inverted propeller that received the liquid and, with the rotation of the drill, worked as a turbine. Then, the liquid was driven by the blades down into the socket. In addition, the device served as a stopper to control the exact drilling depth. Figure 1 presents the main characteristics of this new drill design.

Thus, three experimental groups were formed, as described below:

Control group 1 (Con1): Multiple drill sequence for a conical implant of 10 mm in length and 4.1 mm in diameter, Straumann (Basel, Switzerland): drill diameters were 2.2 mm (used at 800 rpm), 2.8 mm (600 rpm) and 3.5 mm (500 rpm) [11]. 

Control group 2 (Con2): One single drill for a conical implant of 10 mm in length and 4.2 mm in diameter for conical IDAll implant, Implants Diffusion International (Montreuil, France). The speed recommended and used was 1500 rpm. 

**Figure 2:**
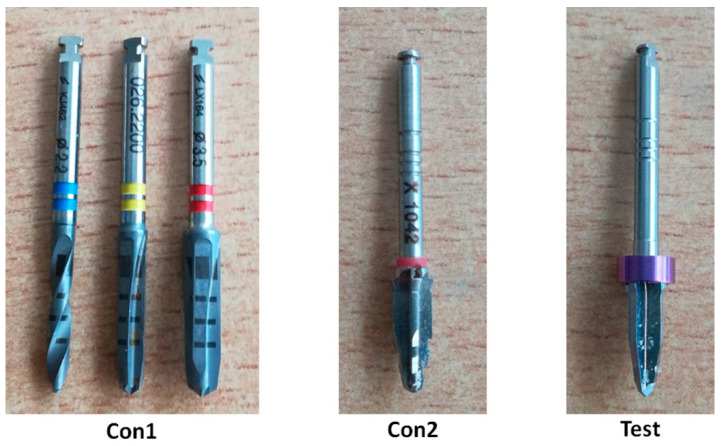
Image of the drill systems used for the osteotomies in the 3 groups.

Test group (Test): One single drill (TURBOdrill^®^) for a conical implant of 10 mm in length and 4.2 mm in diameter for conical IDAll implant, Implants Diffusion International (Montreuil, France). The speed recommended and used was 1500 rpm. Figure 2 shows an image of the drills used for each group.

**Figure 3:**
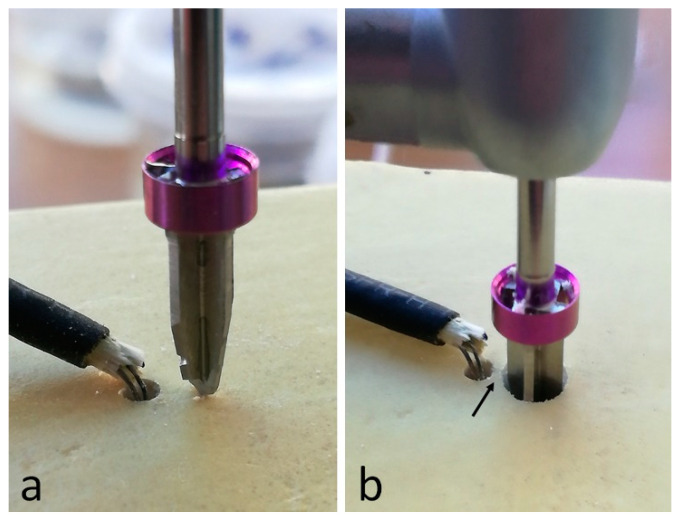
The thermocouple type k positioned in the perforation and the drill positioned before starting the osteotomy (**a**) and after the osteotomy finished (**b**), where the arrow indicates the distance of 1 mm to the sensor.

Three synthetic cortical bone blocks manufactured in polyurethane foam with a density of 40 pounds per cubic foot (PCF), corresponding to 0.62 g/cm^3^ (Nacional Ossos, Jaú, Brazil), were used (one per group). The blocks presented the following dimensions: width of 9.7 cm, height of 5 cm and length of 10 cm. Initially, a perforation to install the sensor was performed with a conical carbide bur at 1 mm in diameter and 3 mm in depth, at a distance previously calculated so that after the osteotomy completed with the proposed system for each group, the final distance between the two perforations was 1 mm. Figure 3 shows these details. 

A type K thermocouple device (Mod. TP-01, Lutron Electronics Co., Inc., Coopersburg, PA, USA) was used for measuring the temperature during the osteotomies, which was coupled to a digital thermometer (Lutron Electronics Co., Inc.) with a resolution of 0.1 °C. Whenever one osteotomy was completed, the next was only started after the temperature returned to the initial level of 18 ± 1 °C (baseline temperature).

For drilling, a drilling machine controlled by an automated system was used, which was used in other previous studies [4,12]. The device controls the milling speed, the load applied during the osteotomy, irrigation volume and intermittent movements. All osteotomies were performed by applying a load of 2 kg, intermittent movements (4 mm, 8 mm and, finishing at 10 mm) and with intense irrigation of 50 mL/min (in condition 2). Irrigation was carried out with distilled water. Then, within these described conditions, twenty osteotomies were performed without irrigation and another 20 with irrigation for each group.

The range of temperature variation was calculated using the maximum temperature value measured and the baseline temperature (ΔT). The data were compared statistically using the ANOVA One-Way test to verify differences between the 3 groups in the two proposed conditions (without and with irrigation). Additionally, Bonferroni’s multiple comparison test was used to determine the individual difference between the 3 groups. All cases where *p* < 0.05 were considered statistically significant. All data were analyzed using GraphPad Prism version 5.01 for Windows (GraphPad Software, San Diego, CA, USA). 

## 3. Results

The measured data of the temperature generated during the osteotomies were collected in an electronic sheet, and the differences between the initial and maximum temperatures were calculated. A normal distribution result was detected of the groups after applied the normality test.

**Figure 4:**
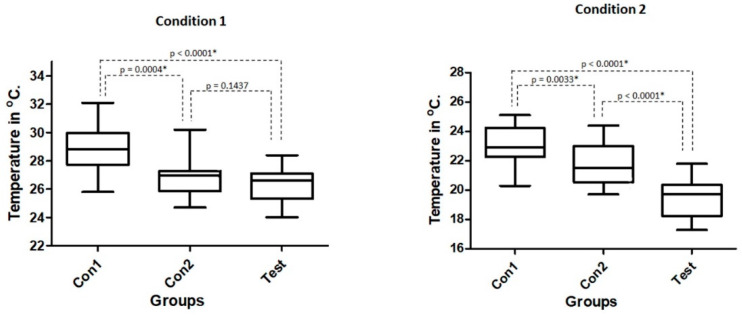
Box Plot graphs presenting the medians, quartiles and ranges of the 3 groups analyzed in both conditions tested (without and with irrigation, respectively) and the statistical comparison between the groups. * shows that they are statistically different.

Significant differences for the measured temperatures during the osteotomies without and with irrigation were detected, in both cases with *p* < 0.0001. Considering absolute values, the Con2 group and Test group (both using one single drill) yielded similar results (not significantly different) in the condition 1 (without irrigation). However, in the Con1 group, significantly higher temperatures were recorded concerning the other 2 groups in both conditions (without and with irrigation). The Box Plot graphs shown in Figure 4 presented the medians, quartiles and ranges of the 3 groups analyzed in both conditions (without and with irrigation) and the statistical comparison between the groups. 

The mean, standard deviation (SD), median and range values of the maximum temperature measured for each group in the 2 conditions proposed are summarized in Table 1. 

**Figure 5:**
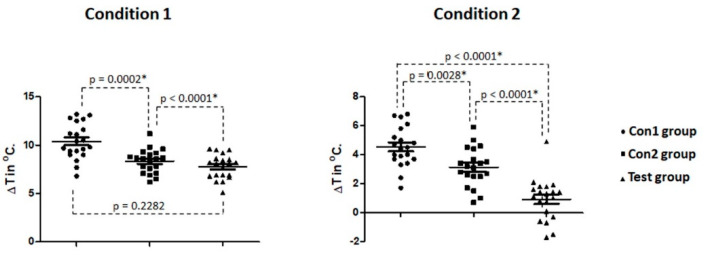
Points graph of the calculated temperature variation (ΔT) between the initial temperature and maximums temperature for each group in both conditions tested and the statistical difference between the groups. * shows that they are statistically different.

The one single drill used in the Con2 and Test groups produced a smaller variation of temperature in comparison with the multiple sequence drills used in the Con1 group, as demonstrated follow the means ± standard deviations concerning baseline values (ΔT). Firstly, the calculated variation of temperature data in the osteotomies without irrigation were: 10.40 ± 1.85 °C for Con1 group; 8.34 ± 1.23 °C for Con2 group; 7.77 ± 1.26 °C for Test group. Whereas, in the osteotomies with irrigation, the calculated values were: 4.54 ± 1.39 °C for Con1 group; 3.14 ± 1.34 °C for Con2 group; 0.93 ± 1.47 °C for Test group. A significant difference was recorded for ΔT between the groups in both conditions (*p* < 0.0001). However, when the groups were compared against each other, only in condition 1 did the Con2 and Test group shows no statistical differences. The calculated values of the temperature variation as well as the statistical comparison between the groups are shown graphically in Figure 5.

Regarding the time required for osteotomy in each group, the average was ~10 s for the Con2 and Test groups, and ~80 s for the Con1 group (including three consecutive drilling sequence plus the time for substitution the drills). The time needed to return to baseline temperature after each implant site preparation procedure was a mean of 10 ± 2 min.

## 4. Discussion

The control of trauma during the handling of peri-implant tissues in surgical procedures for the installation of implants is of fundamental importance to obtain satisfactory results and free of complications. Among the maneuvers performed during surgery to install implants, an osteotomy can be considered the most traumatic step, then this topic has been the subject of several studies and the development of new technologies to minimize the effects of this procedure. In this sense, a new drill design was developed featuring a titanium tube with an internal helix on its stem, which aims to direct the flow of irrigating liquid into the drill blades, improving the cooling of bone tissue during the drilling for osteotomy. Then, our objective was to compare this new drill design with two other drill systems, measuring and comparing the temperature during the drilling procedure performed in a block of synthetic bone. The results showed that the new drill design was more effective in the control of heat production during the osteotomies performed, in comparison with the other two drill systems used as control groups.

This new drill design uses the concept of a single cutter to perform an osteotomy, which, according to previous publications, when compared to conventional drill systems that use a multiple drill sequence, showed a better performance in controlling the temperature [12], and similar healing of bone tissue around the implants installed in prepared beds using a single drill [7,12,13]. In addition to these results from in vitro and preclinical studies in rabbits, a study in humans was presented demonstrating a high success rate (98% of implant survival) with the use of a single drill to install the implants, in which 350 implants were evaluated [14]. Conversely, as described by Li et al. [15], there is a great concern for the risk of heat generation during milling with a single cutter, mainly in higher density bone tissue and for the accumulation of bone chips inside the drill. This accumulation of residues inside the cutting part of the drill and its contact with the side of the drilling will result in additional heat generation [15]. In this sense, the device coupled to the stem of the new drill design, which works as a propeller turbine for the cooling liquid, in addition to increasing the temperature control of the drill blades, can eliminate the bone residues inside of the drill. Still, the intermittent movements applied during the performance of the osteotomy help this elimination of bone residues and are important in controlling the temperature [4]. 

Other authors have reported that the drilling procedures for osteotomy should be minimally traumatic, which would be highly recommended to preserve the bone tissue by preventing damage to its healing potential [16]. In addition, the drilling to perform the surgical bed (osteotomy) for the installation of endosseous implants, produces a large local inflammatory reaction, which can be controlled and/or reduced by the use of adequate irrigation technique [5]. The results obtained in the present study showed a lower temperature rise in the groups where a single drill was used to perform the osteotomy (Con2 group and Test group), in comparison with the group where a drilling system with multiple drills (Con1 group) was used. Comparing the data with irrigation, that is a more similar condition with a clinical scenario, the Test group was 16% < Con1 group and 11% < Con2, whereas the Con2 was 6% < Con1 group.

The manufacture of cutters and their performance is directly related to engineering factors and mechanical functioning. For example, drills with double-positive cutters reduce the cutting pressure, consume less power and create less heat [17]. Then, the test carried out without irrigation (condition 1) serves, mainly, to analyze the efficiency of the different types of cutters. From the results obtained in the present study, when the samples were tested without irrigation, it was demonstrated that the 3 drill systems tested present high quality in their designs and excellent performance since the variation in general average from the initial temperature to final temperature was relatively low. Moreover, the heat generated in the drilling operation is also roughly proportional to the undeformed chip thickness and cutting forces [18]. 

Other authors have described that mechanical factors (drill and blade design, cutting precision, drill diameter) and technical factors (drilling protocol, speed and force applied, drill angulation, irrigation system and torque applied), are important in determining the physical stress generated [19,20]. Then, as the mechanical factors are determined by the manufacturer of the drilling system, it is the technical factors that may vary during its execution, as clinically this will depend directly on the operator. However, in our study, automated equipment was used so that there were no variations and/or errors during the execution of predetermined technical factors for each drilling systems tested. Regards to the technique applied for the groups, only the drill speed was different between them, which followed the recommendation of each manufacturer. In this sense, a variety of propositions were described in the literature [10], however, the drill design (project) must determine the ideal speed for each drilling system. 

Another important factor to note is the characteristics of bone density used in our experiment. The cortical bone presented the mayor density and, as described by Sener et al. (2009) [21], that most heat changes are generated in the most superficial part of this compact bone. Then, the sensor was installed at a depth of 3 mm, although the bone block used had the same density throughout its structure. Still, regarding the drilling time, several authors have described that the drilling time can influence the temperature variation values during osteotomy [19]. In this point, the measured time for drilling in the Con1 group (multiple sequences), obviously was superior due to the need to replace the drills, because it is a sequence of 3 cutters, compared to the groups Con2 and Test that use only one drill. This possibility of performing osteotomy with a low-temperature variation using only one drill may prove beneficial to tissues reducing the local damage as well as the patients’ discomfort. 

Some limitations and clinical care of this in vitro study must be considered, such as the fact that an all-cortical bone model and automated equipment were used to perform osteotomies, as this does not reflect the clinical reality. On the other hand, when analyzing from the point of view of the proposed and tested techniques, the use of a single cutter for the preparation of the implant bed does not allow for direction corrections after its execution, unlike the use of multiple cutters, where it is possible correct any direction error during the passage from one to the other drill sequence. Thus, we can say that the use of a single cutter requires greater precision during its use. Another important observation is that the initial temperature of the specimens in this study was ~18 °C, while in the patient we have an initial temperature of ~37 °C, which gives us a variation limit of ~10 °C. Then, when we calculate the temperature variation values with the corporal temperature, in the Con1 group in the condition 1 (without irrigation), the temperature could exceed the critical limit and be causing bone necrosis (~37 °C + 10.4 °C = ~47.4 °C). This scenario could occur due to a failure of the irrigation during the surgical procedure. However, in the other two groups a with an average temperature variation of 8.1 °C, even without irrigation, the value was below the critical point (~37 °C + 8.1 °C = ~45.1 °C). Still, in all groups when the osteotomies were performed using irrigation the values were far removed from the critical value.

## 5. Conclusions

Within the limitations of the present in vitro study, we can conclude that the single drill with the new design for improving the irrigation and temperature control, demonstrated that the new device coupled to the drills (TURBOdrill^®^) increases and directs the flow of the irrigation liquid and results in better temperature control during the osteotomy, in comparison with the drill designs that use conventional external irrigation.

## Figures and Tables

**Table 1:** Mean, standard deviation (SD), median and range values of the maximum temperature measured for each group in the 2 conditions proposed. Values in centigrade degrees (°C).

Variables	Condition 1			Condition 2		
Groups	Mean and SD	Median	Range Values	Mean and SD	Median	Range Values
Con1	28.9 ± 1.68	28.8	25.8 to 32.1	23.1 ± 1.27	22.9	20.3 to 25.1
Con2	26.9 ± 1.31	27.0	24.7 to 30.2	21.7 ± 1.36	21.5	19.7 to 24.4
Test	26.3 ± 1.28	26.6	24.0 to 28.4	19.4 ± 1.29	19.7	17.3 to 21.8
